# CISepsis: a causal inference framework for early sepsis detection

**DOI:** 10.3389/fcimb.2024.1488130

**Published:** 2024-11-29

**Authors:** Qiang Li, Dongchen Li, He Jiao, Zhenhua Wu, Weizhi Nie

**Affiliations:** ^1^ School of Microelectronics, Tianjin University, Tianjin, China; ^2^ School of Pharmaceutical Science and Technology, Tianjin University, Tianjin, China; ^3^ Department of Cardiovascular Surgery Intensive Care Unit, Tianjin Chest Hospital, Tianjin, China; ^4^ School of Electrical and Information Engineering, Tianjin University, Tianjin, China

**Keywords:** sepsis, MIMIC-IV, causal inference, back-door intervention, instrumental variable

## Abstract

**Introduction:**

The early prediction of sepsis based on machine learning or deep learning has achieved good results.Most of the methods use structured data stored in electronic medical records, but the pathological characteristics of sepsis involve complex interactions between multiple physiological systems and signaling pathways, resulting in mixed structured data. Some researchers will introduce unstructured data when also introduce confounders. These confounders mask the direct causality of sepsis, leading the model to learn misleading correlations. Finally, it affects the generalization ability, robustness, and interpretability of the model.

**Methods:**

To address this challenge, we propose an early sepsis prediction approach based on causal inference which can remove confounding effects and capture causal relationships. First, we analyze the relationship between each type of observation, confounder, and label to create a causal structure diagram. To eliminate the effects of different confounders separately, the methods of back-door adjustment and instrumental variable are used. Specifically, we learn the confounder and an instrumental variable based on mutual information from various observed data and eliminate the influence of the confounder by optimizing mutual information. We use back-door adjustment to eliminate the influence of confounders in clinical notes and static indicators on the true causal effect.

**Results:**

Our method, named CISepsis, was validated on the MIMIC-IV dataset. Compared to existing state-of-the-art early sepsis prediction models such as XGBoost, LSTM, and MGP-AttTCN, our method demonstrated a significant improvement in AUC. Specifically, our model achieved AUC values of 0.921, 0.920, 0.919, 0.923, 0.924, 0.926, and 0.926 at the 6, 5, 4, 3, 2, 1, and 0 time points, respectively. Furthermore, the effectiveness of our method was confirmed through ablation experiments.

**Discussion:**

Our method, based on causal inference, effectively removes the influence of confounding factors, significantly improving the predictive accuracy of the model. Compared to traditional methods, this adjustment allows for a more accurate capture of the true causal effects of sepsis, thereby enhancing the model's generalizability, robustness, and interpretability. Future research will explore the impact of specific indicators or treatment interventions on sepsis using counterfactual adjustments in causal inference, as well as investigate the potential clinical application of our method.

## 1 Introduction

Sepsis is a severe immune response to infection that can lead to tissue damage and organ dysfunction. This response can progress to septic shock, including organ failure and extremely low blood pressure Leone (2016). Its hallmark is an immune system response imbalance, with high incidence and mortality rates Inkpen et al. (2023). Despite significant advances in medical technology and treatment methods, the diagnosis and treatment of sepsis remain among the important challenges faced by clinicians. Convincing evidence suggests that every hour of antibiotic delay significantly increases the mortality rate associated with sepsis Ferrer et al. (2014). In the actual treatment process, healthcare professionals such as doctors, nurses, and pharmacists may experience delays in communication due to unclear symptoms presented by patients, potentially leading to delayed treatment for subsequent sepsis Goh et al. (2021). Therefore, early prediction of sepsis onset to arrange and implement a sepsis treatment plan is crucial. Timely treatment can effectively reduce mortality and the occurrence of complications.

The large number of instruments in the ICU can produce highly granular data, and this high-quality database constitutes a cornerstone for integrating AI into clinical practice Li et al. (2024). Currently, some popular early sepsis prediction models are primarily based on structured data, which is a multivariate time series. While some models have shown good performance, traditional prediction models may easily learn false correlations. These false correlations may point to information unrelated to the disease but related to the outcome, compromising the model’s generalization capability and robustness Li et al. (2021). At the same time, there is missing information in the data set, and a lot of work has been devoted to solving the problem of missing data Apalak and Kiasaleh (2022); Wang and Yao (2021); Zabihi et al. (2019); Singh et al. (2019a). To address this natural deficiency, some researchers also utilize unstructured data from electronic health records such as clinical texts and radiological images. A large number of studies have focused on the impact of a specific metric on sepsis Gao et al. (2022); Jiang et al. (2024); Yang et al. (2023). However, the pathological characteristics of sepsis involve complex interactions between multiple physiological systems and signaling pathways He et al. (2022). Confounding occurs when the association between a presumed cause *X* and its observed effect *Y* is actually due to a common cause *C* Sun et al. (2022). This *C* is the confounder. There is a confounder in the structured data, and the introduction of multimodal data introduces another confounder that masks direct causal relationships from *X* to *Y*, leading the model to learn misleading correlations and unreliable model generalization. Our motivation is to eliminate the influence of confounders in the data and learn real causal effects to improve the performance and generalization ability of the model. Causal inference is often used to solve the problem of confounding factors in data. Liu et al. improve reasoning ability by retaining good bias and mitigating bad bias (Liu et al., 2023). Influenced by the work of Zhang Chang et al. (2023), Song Song et al. (2024), we propose an early sepsis prediction model based on Causal Inference, which can learn the true and effective causal relationship by eliminating the influence of confounders, and improving the model performance and robustness. We propose a Feature Extraction Module to model Instrumental Variables, using auxiliary variables to eliminate the effect of confounders that are difficult to observe. Instrumental variables and distractors are learned from clinical indicators. We propose a Mutual Information Optimization Module to constrain the Instrumental Variable to ensure that it is an effective IV. We propose a Causal Learning Module based on Back-door Adjustment, which cuts off the back-door path to eliminate the influence of observable confounders in the data and learn the true causal effect. The prior knowledge is used to define the influence effect of two independent confounder factors on causal features so that the model can approximately learn the true causal effect, and improve the robustness and generalization ability of the model.

## 2 Materials and method

### 2.1 Dataset

We used the Medical Information Mart for Intensive Care (MIMIC-IV) dataset Johnson et al. (2023). MIMIC-IV is a publicly available database sourced from the electronic health record of the Beth Israel Deaconess Medical Center. It covers hospital admissions data from 2008 to 2019. One of the authors of this study(DL) has completed the Collaborative Institutional Training Initiative examination (Certification number: 60594470).

### 2.2 Data collection

MIMIC-IV contains a total of 53,150 patients, 69,211 hospital admission records, and 76,540 records of intensive care unit stays Nie et al. (2023). In the Third International Consensus Definition for Sepsis and septic shock (Sepsis-3), sepsis is defined as a patient’s SOFA score ≥ 2 after suspicion of infection Singer et al. (2016). Our primary focus is on early sepsis prediction among patients admitted to the ICU, using data collected after ICU admission. The sepsis is defined as positive samples, while the non-sepsis is considered as negative samples. Based on this, we extracted all available records of ICU admissions and further refined the dataset using the following criteria: (1) Patients who were at least 18 years old at their first hospital admission. (2) Only one ICU admission per hospital stay.

### 2.3 Preliminaries

**Figure 1:**
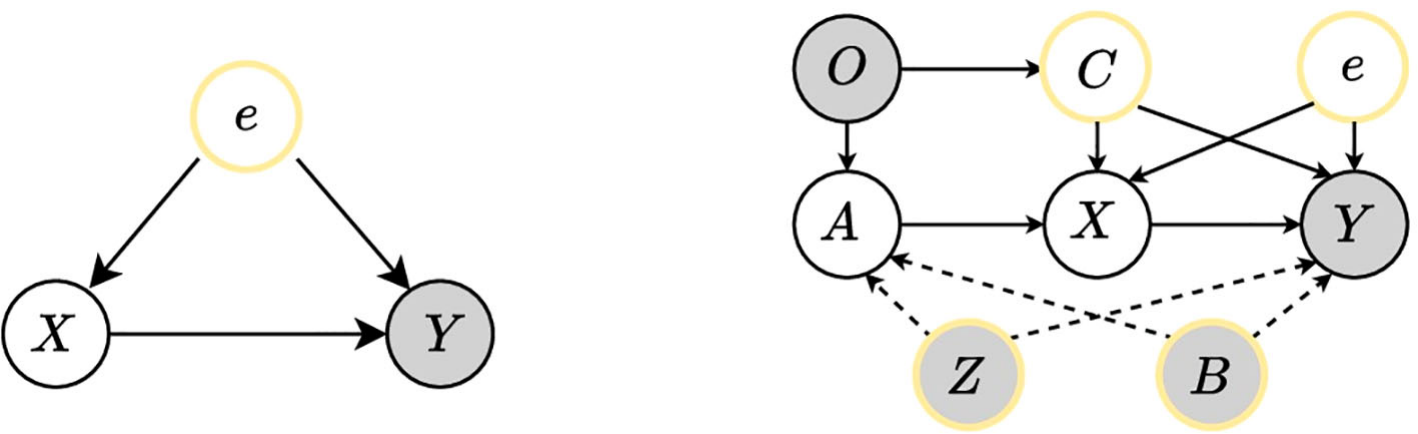
The causal DAG proposed by our method. The gray circles represent the observed values, the yellow circles represent confounders, and the white circles represent the intermediate variables in the model. The left shows the influence of the confounder *e* on the true causality *X* → *Y*, and the right shows the causal DAG of our proposed method, where we introduce Instrumental Variables and Back-door Intervention methods to eliminate the influence of the confounders and learn the true causality.

The early prediction of sepsis can be regarded as a classification task. As the left in **Figure 1** shows, the traditional model thinks that this task has two difficulties, one is how to accurately extract features *X* from a lot of data, and the other is how to accurately predict the disease label *Y* from the extracted features *X*. But there is a confounder *e*, *e* is the error term containing the unobserved potential that affects *X* and *Y*. We assume the confounder *e* is with zero expectation and finite variance, then the relationship between *X* and *Y* is *Y* = *f*(*X*) + *e*. For the path from *X* to *Y*, if the nodes in the set *e* are not descendants of *X*, and the *e* will block all back-door paths between *X* and *Y*, then *e* satisfies the back-door criterion of (*X, Y*), and all back-door paths can be cut off by intervening in the set *e*, which is the back-door adjustment. Both the front-door adjustment and the back-door adjustment are ways to eliminate the effects of confounders by intervening to cut off the back-door path. The difference is that back-door adjustment can only be used when the confounder is observable; if the confounder cannot be represented or is not observable, the front-door adjustment is needed by adding an auxiliary variable to the front-door path. A valid Instrumental Variable (IV) *A* should satisfy three conditions Bennett et al. (2019); Hartford et al. (2017); Singh et al. (2019b); Yuan et al. (2022):

1. ℙ(*X*|*A*) ≠ ℙ(*X*), *A* is related to *X*.
2. ℙ(*Y* |*A,X,e*) = ℙ(*Y* |*X,e*), *A* does not directly affect *Y*.
3. 𝔼(*e*|*A*) = 𝔼(*e*), *A* should be unconfounded.

We introduce the instrumental variable *A* to eliminate the effect of the confounder *e*. In addition, if there is an exogenous variable *C* (ℙ(*e*|*C*) = ℙ(*e*)) Yuan et al. (2022), we can use it to make a more accurate estimation. There are not only unobserved confounder *e* in the data but also observed confounder note *Z* and static indicator *B*. They are introduced at the same time as IV is introduced. *A* can be regarded as features extracted from observed data, while descriptions of these features exist in the note. Static indicators have prior probabilities that affect both *A* and *Y*, as the right in **Figure 1**. Assuming that these two confounders, *e*, and *C* are independent of each other after the instrumental variables eliminate the effects of *e* and *C*, all back-door paths from *X* to *Y* can be blocked by back-door adjustment, removing the impact of *Z* and *B*. By analyzing the data, we design a causal Directed Acyclic Graph (DAG) as shown in **Figure 1** and eliminate the influence of confounders through instrumental variables and back-door adjustment. Our method is named as CISepsis.

**Figure 2:**
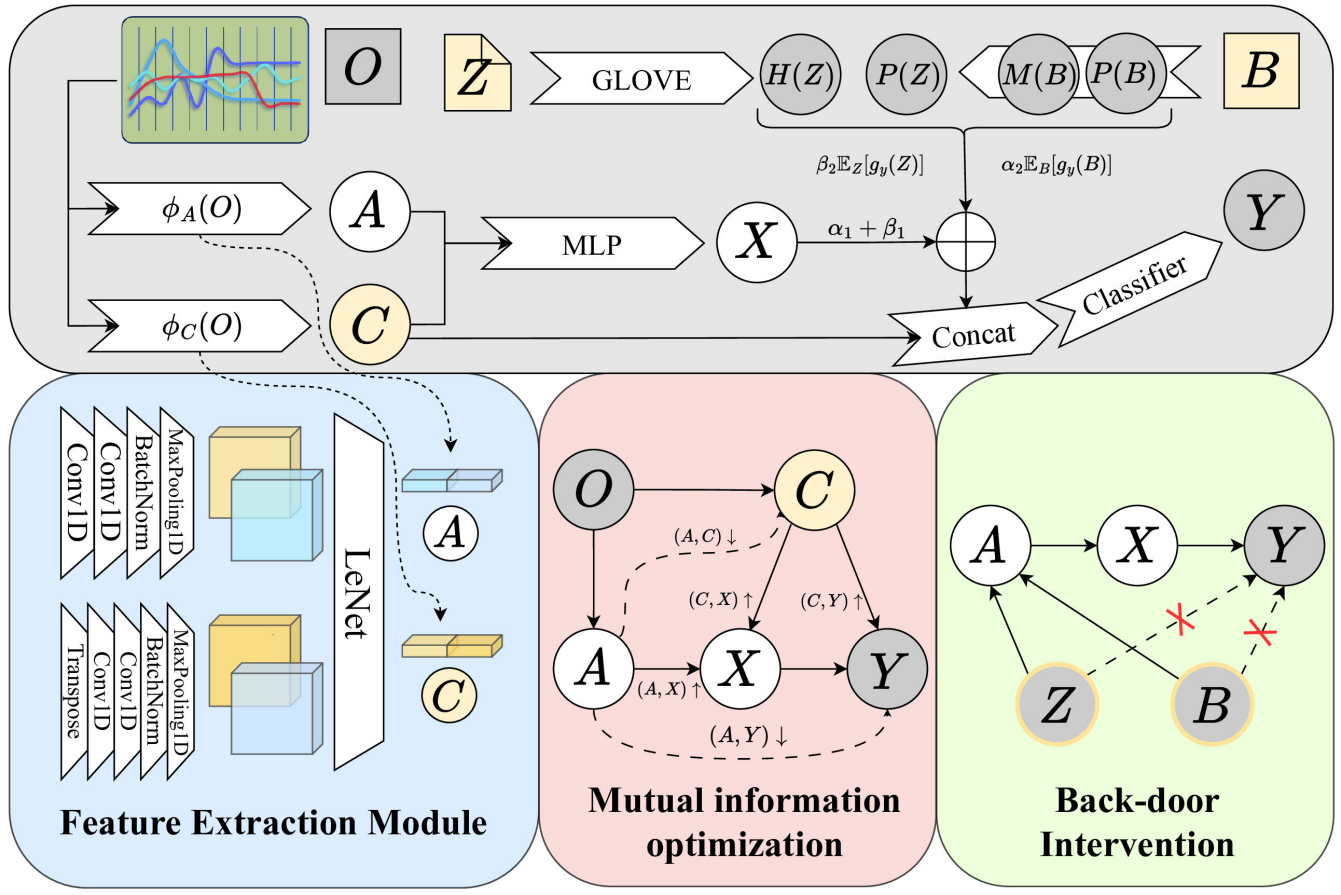
The overall framework of our proposed method. Firstly, we extract features from the observed data *O* to obtain the instrumental variable *A* and the confounder *C*, then let *A* be a valid instrumental variable by optimizing the mutual information. Finally blocked all back-door paths by back-door adjustment.

In this paper, we propose an early sepsis prediction method based on causal inference, specifically utilizing the instrumental variable and back-door adjustment, as shown in **Figure 2**. Our method mainly includes the generation of the instrumental variable, the use of mutual information to optimize the learning of the instrumental variable, and the use of back-door adjustment to eliminate the influence of confounders. We present the detailed implementations of our method in this section.

### 2.4 Modeling instrumental variable

We designed a feature extraction module to model the Instrumental Variables, as shown in the blue square in **Figure 2**. The instrumental variable *A* is first generated from the clinically observed various types of indicators *O*. Similar to previous work Bennett et al. (2019); Hartford et al. (2017); Singh et al. (2019b); Yuan et al. (2022), assuming 𝔼(*e*|*A*) = 𝔼(*e*), *O* is an exogenous variable, then the instrumental variable *A* and the confounder *C* are also exogenous, satisfying the third condition stated in the previous chapter. Define the networks that generate *A* and *C* as *ϕ_A_* (·) and *ϕ_C_* (·). Suppose the size of *O* is (*t,o*), where *t* represents the time dimension and *o* represents the number of features. On the one hand, *O* through a 1D convolution with input channel *t*, output channel 64, kernel size 3, padding 1, transposed and then through a 1D convolution with input channel *o*, output channel 32, kernel size 3, padding 1. The convolution result is obtained by a BatchNorm layer and a MaxPooling layer, then the feature map of size (32, 32) is obtained; On the other hand, *O* through a 1D convolution with input channel *o*, output channel 64, kernel size 3, padding 1 after transposed, then through another 1D convolution with input channel *t*, output channel 32, kernel size 3, padding 1 after transposed. The convolution result is also passed through a BatchNorm layer and a MaxPooling layer to obtain the feature map of size (32, 32). The tensor of (1,64) is obtained after the feature maps are passed through LeNet, and the encoding result is obtained after concatenating them together. The generation of *A* and *C* uses the same network, and the final size is both (1, 128). After learning the instrumental variable *A* from the observed data *O* we have:


(1) E[Y|A]=E[g(X)|A]=∫g(X)dℙ[X|A]

We first estimate *g*(·) by learning ℙ[*X*|*A*]. The causal feature *X* is generated by concatenating *A* and *C* together through a multilayer perceptron(MLP).

### 2.5 Mutual information optimization learning

For *A* to be a valid IV, we also need to satisfy the first two conditions of the previous chapter, making *ϕ_A_* (*O*) related to *X* and conditionally independent of *Y*, in addition, making *ϕ_C_* (*O*) related to *X* and *Y*. This process proceeds through the process of optimizing mutual information. For example, let *ϕ_A_* (*O*) and *X* be correlated, we sample from the distribution of *ϕ_A_* (*O*) and *X*, improve the correlation of *ϕ_A_* (*O*) and *X* by maximizing the mutual information between them. Based on our proposed causal DAG, we design the mutual information optimization module in the pink square in **Figure 2**, which is divided into the following steps:

Maximize the mutual information of *ϕ_A_* (*O*) and *X*. To maximize the mutual information, we first study variational distribution *q*(*X*|*ϕ_A_* (*O*)) to approximate ℙ(*X*|*ϕ_A_* (*O*)). Inspired by previous research Yuan et al. (2022); Cheng et al. (2020); Oord et al. (2018), we define the variational distribution and log-likelihood loss with network parameters *θ_AX_* as:


(2) $$logq_{\theta_{AX}}(X|\phi_{A}\left(O\right))=-\frac{{\left(X-\mu_{\theta_{AX}}\right)}^{2}}{e^{\sigma_{\theta_{AX}}}}-\sigma_{\theta_{AX}}$$

(3) $${ℒ}_{AX}^{LLL}=-\frac{1}{d}\sum_{i=1}^{d}logq_{\theta_{AX}}(x_{i}|\phi_{A}\left(O{)}_{i}\right)$$

where $\mu_{\theta_{AX}}$ and $\sigma_{\theta_{AX}}$ represent the mean and log variance, *d* represents the number of samples. Sampling *ϕ_A_* (*O*)*_i_* and *x_i_* from *ϕ_A_* (*O*) and *X*. Define the variational distribution as *q*(*x_i_* |*ϕ_A_* (*O*)*_i_*) for the positive sample pair, *q*(*x_j_* |*ϕ_A_* (*O*)*_i_*) for the negative sample pair, where *i* ≠ *j*. After minimizing Equation 3, we obtain the optimal distribution of $q_{\theta_{AX}}(X|\phi_{A}\left(O\right))$ with parameter *θ_AX_*. To maximize the difference between positive and negative sample pairs and improve the correlation between *ϕ_A_* (*O*) and *X*, we minimize the following equation:


(4) $$\begin{array}{l} {ℒ}_{AX}=-\frac{1}{d^{2}}\sum_{i=1}^{d}\sum_{j=1}^{d}(logq_{\theta_{AX}}(x_{i}|\phi_{A}\left(O{)}_{i}\right)-logq_{\theta_{AX}}(x_{j}|\phi_{A}\left(O{)}_{i}\right)) \end{array}$$

Minimize the mutual information of *ϕ_A_* (*O*) and *Y*. To minimize the mutual information, we first study variational distribution *q*(*Y*|*ϕ_A_* (*O*)) to approximate ℙ(*Y*|*ϕ_A_* (*O*)). Similarly, the network parameters are defined as *θ_AY_*. The log-likelihood loss function for *q*(*Y* |*ϕ_A_* (*O*)) is given as:


(5) $${ℒ}_{AY}^{LLL}=-\frac{1}{d}\sum_{i=1}^{d}logq_{\theta_{AY}}(y_{i}|\phi_{A}\left(O{)}_{i}\right)$$

Sampling *ϕ_A_* (*O*)*_i_* and *y_i_* from *ϕ_A_* (*O*) and *Y*. Define the variational distribution as *q*(*y_i_* |*ϕ_A_* (*O*)*_i_*) for the positive sample pair, *q*(*y_j_* |*ϕ_A_* (*O*)*_i_*) for the negative sample pair, where *i* ≠ *j*. After minimizing Equation 5, we obtain the optimal distribution of $q_{\theta_{AY}}(Y|\phi_{A}\left(O\right))$ with parameter *θ_AY_*. To minimize the difference between positive and negative sample pairs and reduce the correlation between *ϕ_A_* (*O*) and *Y*, we minimize the following equation:


(6) $$\begin{array}{l} {ℒ}_{AY}=\frac{1}{d^{2}}\sum_{i=1}^{d}\sum_{j=1}^{d}(logq_{\theta_{AY}}(y_{i}|\phi_{A}\left(O{)}_{i}\right)-logq_{\theta_{AY}}(y_{j}|\phi_{A}\left(O{)}_{i}\right)) \end{array}$$

With these two steps, we satisfy the first two conditions in the previous chapter such that *A* becomes a valid IV. Furthermore, we need to optimize the mutual information between the confounding *C* and the other variables.

Maximize the mutual information of *ϕ_C_* (*O*) with *X* and *Y*. When generating the instrumental variable *A*, we used the same network to generate an exogenous variable *C*, defined as the confounder in *O*, related to *X* and *Y*. Similar to the process used to maximize the mutual information between *ϕ_A_* (*O*) and *X*, we first study variational distribution *q*(*X*|*ϕ_C_* (*O*)) and *q*(*Y* |*ϕ_C_* (*O*)) to approximate ℙ(*X*|*ϕ_C_* (*O*)) and ℙ(*Y* |*ϕ_C_* (*O*)). The log-likelihood loss functions are given as:


(7) $${ℒ}_{CX}^{LLL}=-\frac{1}{d}\sum_{i=1}^{d}logq_{\theta_{CX}}(x_{i}|\phi_{C}\left(O{)}_{i}\right)$$

(8) $${ℒ}_{CY}^{LLL}=-\frac{1}{d}\sum_{i=1}^{d}logq_{\theta_{CY}}(y_{i}|\phi_{C}\left(O{)}_{i}\right)$$

To maximize the difference between positive and negative sample pairs and improve the correlation between *ϕ_C_* (*O*) and *X,Y*, we minimize the following equations:


(9) $$\begin{array}{l} {ℒ}_{CX}=-\frac{1}{d^{2}}\sum_{i=1}^{d}\sum_{j=1}^{d}(logq_{\theta_{CX}}(x_{i}|\phi_{C}\left(O{)}_{i}\right)-logq_{\theta_{CX}}(x_{j}|\phi_{C}\left(O{)}_{i}\right)) \end{array}$$

(10) $$\begin{array}{l} {ℒ}_{CY}=-\frac{1}{d^{2}}\sum_{i=1}^{d}\sum_{j=1}^{d}(logq_{\theta_{CY}}(y_{i}|\phi_{C}\left(O{)}_{i}\right)-logq_{\theta_{CY}}(y_{j}|\phi_{C}\left(O{)}_{i}\right)) \end{array}$$

Minimize the mutual information of *ϕ_A_* (*O*) and *ϕ_C_* (*O*). To minimize the mutual information, we first study variational distribution *q*(*ϕ_C_* (*O*)|*ϕ_A_* (*O*)) to approximate ℙ(*ϕ_C_* (*O*)|*ϕ_A_* (*O*)). Similarly, the network parameters are defined as *θ_AC_*. The log-likelihood loss function for *q*(*ϕ_C_* (*O*)|*ϕ_A_* (*O*)) is given as:


(11) $${ℒ}_{AC}^{LLL}=-\frac{1}{d}\sum_{i=1}^{d}logq_{\theta_{AC}}(\phi_{C}{\left(O\right)}_{i}|\phi_{A}\left(O{)}_{i}\right)$$

Sampling *ϕ_A_* (*O*)*_i_* and *ϕ_C_* (*O*)*_i_* from *ϕ_A_* (*O*) and *ϕ_C_* (*O*). Define the variational distribution as *q*(*ϕ_C_* (*O*)*_i_* |*ϕ_A_* (*O*)*_i_*) for the positive sample pair, *q*(*ϕ_C_* (*O*)*_j_* |*ϕ_A_* (*O*)*_i_*) for the negative sample pair, where *i* ≠ *j*. After minimizing Equation 11, we obtain the optimal distribution of $q_{\theta_{AC}}(\phi_{C}\left(O\right)|\phi_{A}\left(O\right))$ with parameter *θ_AC_*. To minimize the difference between positive and negative sample pairs and reduce the correlation between *ϕ_A_* (*O*) and *ϕ_C_* (*O*), we minimize the following equation:


(12) $$\begin{array}{l} {ℒ}_{AC}=\frac{1}{d^{2}}\sum_{i=1}^{d}\sum_{j=1}^{d}(logq_{\theta_{AC}}(\phi_{C}{\left(O\right)}_{i}|\phi_{A}\left(O{)}_{i}\right)-logq_{\theta_{AC}}(\phi_{C}{\left(O\right)}_{j}|\phi_{A}\left(O{)}_{i}\right)) \end{array}$$

In the training process, we first need to minimize Equations 3, 5, 7, 8 and 11 to obtain the best parameter *θ_AX_*, *θ_AY_*, *θ_CX_*, *θ_CY_* and *θ_AC_*. The mutual information is optimized after the best parameters are obtained by minimizing Equations 4, 6, 9, 10 and 12.


(13) $${ℒ}^{LLL}={ℒ}_{AX}^{LLL}+{ℒ}_{AY}^{LLL}+{ℒ}_{CX}^{LLL}+{ℒ}_{CY}^{LLL}+{ℒ}_{AC}^{LLL}$$

(14) $${ℒ}_{1}={ℒ}_{AX}+{ℒ}_{AY}+\delta_{1}\left({ℒ}_{CX}+{ℒ}_{CY}\right)+\delta_{2}{ℒ}_{AC}$$

The best network parameters are obtained by minimizing Equations 13, 14, where *δ*_1_ and *δ*_2_ in Equation 14 are hyperparameters that can be trained.

### 2.6 Back-door adjustment

Note *Z* and static information *B* can be observed in the patient’s data. As shown in the ‘Back-door Intervention’ in **Figure 2**, to learn the true causal effect of *X* → *Y*, we propose a causal learning module based on Back-door Adjustment to eliminate the effects of *Z* and *B*. Sepsis is more likely to occur in patients with trauma, chronic diseases, or low immunity, among which the elderly, pregnant women, and infants are more susceptible to infection. This leads to a prior probability in *B* that affects both *A* and *Y*, and a back-door path exists *X* ← *A* ← *B* → *Y*; However, the clinical note documents the various aspects of a patient’s condition, not only related to the result(disease or not) but also related to various physical indicators of the patient, which also affects *A* and *Y*, there is also a back-door path *X* ← *A* ← *Z* → *Y*. Due to the existence of back-door paths, the model learns false associations. If the back-door path is cut off, the effect of the confounder is eliminated and the model can learn the true causal impact of *X* → *Y*. Let *W* = {*Z,B*}, in the absence of intervention, *P*(*Y* |*X*) can be expressed as:


(15) $$P(Y|X)=\sum_{W}P(Y|X,W)P(W|X)$$

Pearl first proposed using the back-door adjustment method to remove the influence of confounding factors, and the *do*-operator realizes the back-door adjustment. Intervening on *X*, since *Z* and *B* are independent, we have:


(16) $$\begin{array}{l} P(Y|do\left(X\right))=\sum_{b}P(Y|X,B=b)P\left(B=b\right)+\sum_{z}P(Y|X,Z=z)P\left(Z=z\right) \end{array}$$

The final layer of the prediction network for binary classification is the Softmax layer. After averaging the confounding effects we have the following:


(17) $$\begin{array}{l} \sum_{b}P(Y|X,B)P(B)=\sum_{b}\left[Softmax(f_{y}(X,B))]P(B\right)=E_{B}[Softmax(f_{y}(X,B))]\\ \;\overset{NWGM}{\approx }Softmax(E_{B}[f_{y}(X,B)])\\ \sum_{z}P(Y|X,Z)P(Z)=\sum_{z}\left[Softmax(f_{z}(X,Z))]P(Z\right)=E_{Z}[Softmax(f_{z}(X,Z))]\\ \;\overset{NWGM}{\approx }Softmax(E_{Z}[f_{z}(X,Z)]) \end{array}$$

Direct calculation requires relatively large sampling calculation cost, so Normalized Weighted Geometric Mean(NWGM) is used to do approximation in Equation 17, and the expectation is integrated into the network. In our approach, we assume that:


(18) $$\begin{array}{ll} f_{y}\left(X,B\right) & =\alpha_{1}X+\alpha_{2}\sum_{B}P\left(B\right)M\left(B\right)\\ f_{y}\left(X,Z\right) & =\beta_{1}X+\beta_{2}\sum_{Z}P\left(Z\right)⊙\frac{H\left(Z\right)X^{T}}{n}⊙H\left(Z\right) \end{array}$$

Where *α*_1_ *, α*_2_ *, β*_1_ and *β*_2_ are hyperparameters that can be learned. *P*(*B*) represents the prior probability of each feature in the static indicators, and *M*(*B*) represents the influence effect corresponding to each feature for *X*, encoded to the same dimension as *X* through a fully connected layer. *P*(*Z*) represents the prior probability of each word, and *H*(*Z*) represents the vector representation of each word. Using only texts from the ICU period, *P*(*Z*) and *H*(*Z*) are obtained by GLOVE. $\frac{H\left(Z\right)X^{T}}{n}$ represents the importance of each word for *X*, *n* denotes the feature dimension in which each word is encoded, which coincides with *X*, ⊙ represents matrix dot multiplication. Take Equation 18 into Equation 17:


(19) $$\begin{array}{l} P(Y|do\left(X\right))=Softmax\left(E_{B}\left[f_{y}\left(X,B\right)\right]+E_{Z}\left[f_{z}\left(X,Z\right)\right]\right) \end{array}$$

We also need to consider *C* when using instrumental variables. Although *C* represents the confounding factor in the data, it comes from the clinical observation data, and there is still a lot of information that can be used as additional information for the final prediction. In our method, after fully considering the influence effect of confounders, the modified *X* and *C* are concatenated to enter the final classification model together. The previous process eliminated the confounding effect, so the classification model consists of only a few consecutive fully connected layers and activation functions.

## 3 Result

### 3.1 Experiment result

The task of early disease prediction is a sample imbalance task, the number of negative samples (no disease, 0) is much more than the number of positive samples (disease, 1). We divide the dataset into three parts: training set, test set, and validation set, in which the number of positive and negative samples in the training set is equal, while the ratio of negative samples to positive samples in the test set and validation is close to 4:1. We selected the following methods for comparative experiments:(1)EXtreme Gradient Boosting(XGBoost), a decision tree-based machine learning algorithm, suitable for classification and regression problems, has been used by many researchers for early sepsis prediction (Pettinati et al. (2020); Hu et al. (2022); Barton et al. (2019). (2) LSTM, a commonly used time series model, is also used by many researchers for early sepsis prediction (Fagerström et al. (2019); Kaji et al. (2019). (3) MGP-AttTCN, a model that combines joint multi-task Gaussian processes and attention-based deep learning (Rosnati and Fortuin (2021). CNN-LSTM, a combination of convolutional neural networks and LSTM aimed at surpassing current sepsis knowledge limitations (Lauritsen et al. (2020).

**Figure 3:**
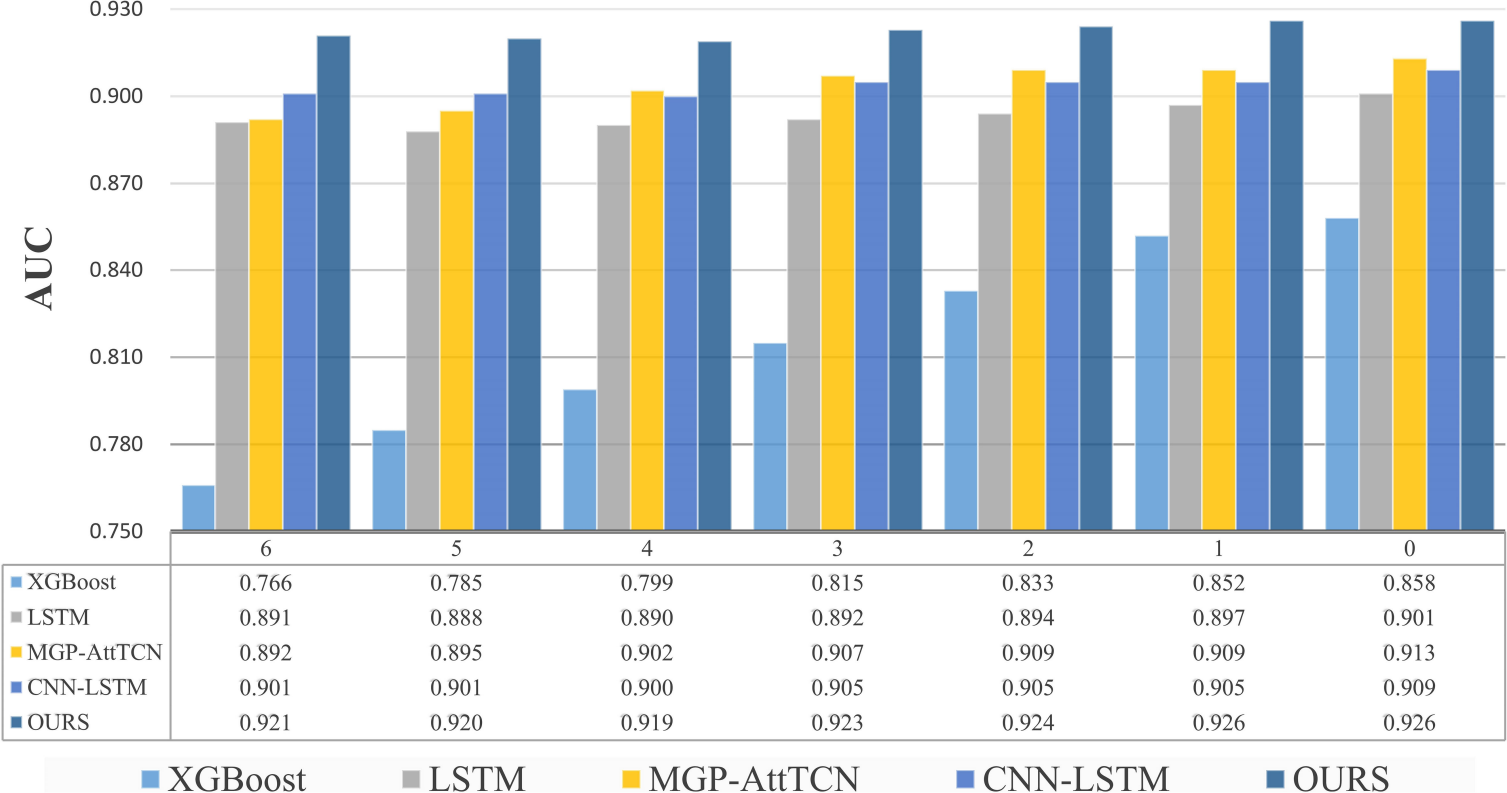
Results of comparative experiments several hours before the onset of sepsis. The ordinate of the upper bar chart represents the value of the indicator AUC, and the abscissa represents the task moment several hours before the onset of sepsis. Below is the specific data table, our method is better than the comparison methods.

A total of seven tasks were included in the comparison experiment: early prediction 0 to 6 hours before the onset of sepsis. The results are shown in **Figure 3**. The bar graph clearly shows that our method is better than the comparison test. The performance of XGBoost is the worst among all models, XGBoost is an ensemble learning method based on the decision tree. In the decision process of the tree model, each feature is used independently, which can be regarded as predicting based on the distribution of data, ignoring the relationship between the features and losing a lot of information. Medical data has complex relationships between features, XGBoost works well with a small number of features, but with a large number of features, it doesn’t perform as well as neural networks. LSTM captures the dependencies in the time series and identifies the context information through the gating mechanism and cell state, but it uses the vector at each time as a whole, also ignores the relationship between each feature, and loses spatial information. As the time interval between the task and the onset of sepsis decreases, all methods show better and better performance. Although some deep learning models have shown good performance, they ignore the spurious correlations in the data, which is not conducive to the generalization and robustness of the model. MGP-AttTCN and CNN-LSTM consider the relationship between features through convolution. However, as the traditional prediction methods, in the process of model training, they only want to learn the content related to the result in the data to improve performance, don’t care whether this part of the content is real causal features. In order to eliminate the influence of confounding factors, we use the causal inference method to eliminate the confounding effect and learn the real causal effect. At the same time, we designed a special encoding module to fully learn the temporal information and spatial information in the data, which has a significant performance improvement compared with the baseline and improves the generalization ability.

### 3.2 Ablation experiment

**Figure 4:**
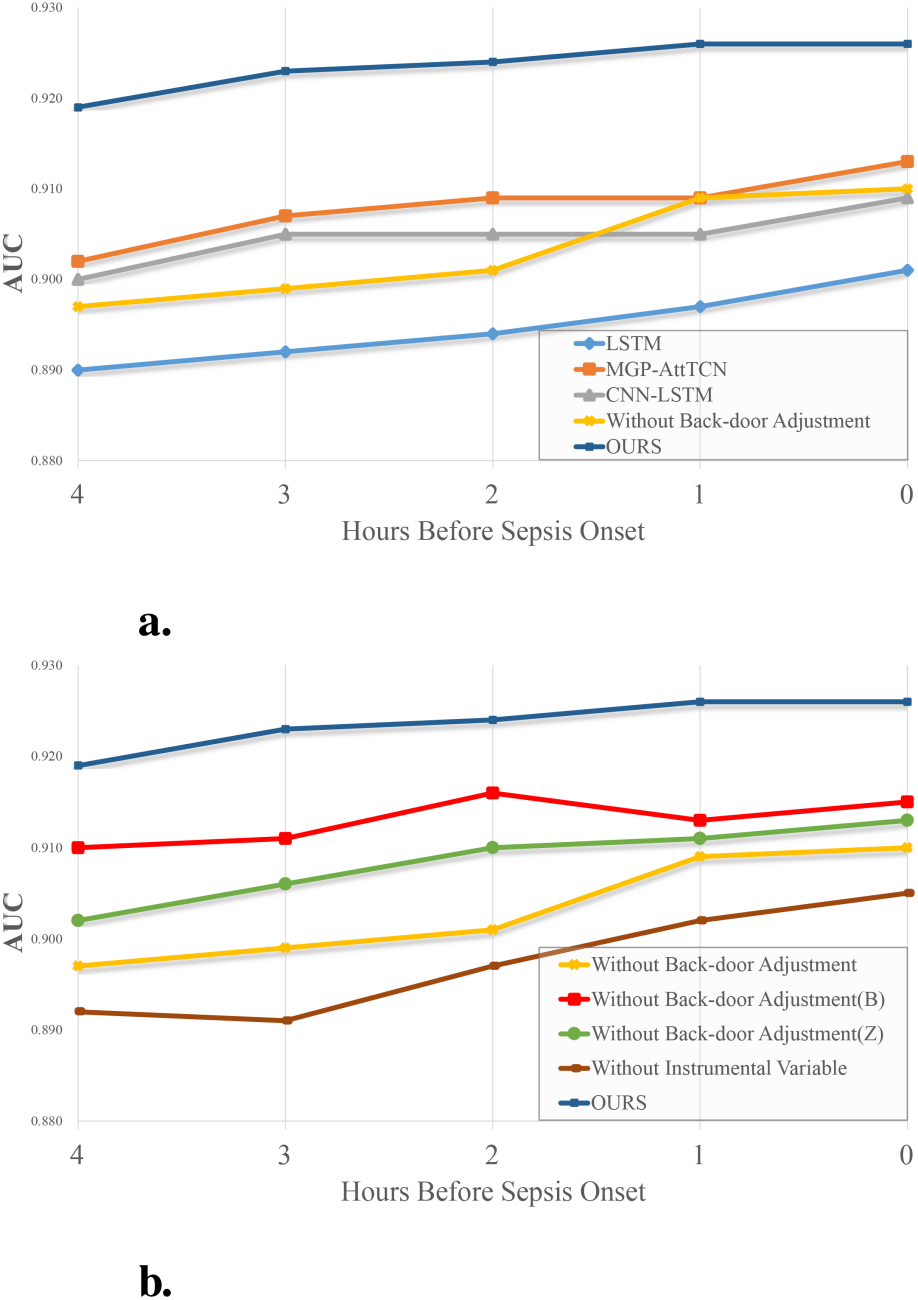
**(A)** Experimental results and **(B)** ablation experiment results. The vertical axis represents the metric AUC, the horizontal axis represents the time between each task and the onset of sepsis.

To further confirm the effectiveness of our method, we designed such ablation experiments: Keep only the part that uses instrumental variables, and remove the modules that use back-door adjustment. Keep the part that uses instrumental variables and keep the data *Z* in the back-door adjustment; Keep the part that uses instrumental variables, and keep the data in the back-door adjustment *B*; Only the part that uses the back-door adjustment is kept, and the instrumental variable part only extracts the feature *X* using our feature extraction module. The results are shown in **Table 1**. It can be intuitively found in **Figure 4A** that the performance of our method is better than that of the comparison method. At the same time, if only the instrumental variable part is retained in our method, a good performance is still obtained, which indicates that the instrumental variable generated by us effectively eliminates the confounders in the observed data *O* and the influence of the confounders that are difficult to observe in the system. Although it is slightly worse than several contrast trials, this is because in this experiment only the observation data *O* is used, but not *B* and *Z*, thus a lot of information is lost.

**Table 1:** Changing components in our model.

Components(Data used)			4	3	2	1	0
Instrumental Variable (*O*)	Back-door Adjustment (*Z*)	Back-door Adjustment (*B*)	AUC	AUC	AUC	AUC	AUC
✓ ✓ ✓ ✓	✓ ✓ ✓	✓ ✓ ✓	0.897 0.910 0.902 0.892 **0.919**	0.899 0.911 0.906 0.891 **0.923**	0.901 0.916 0.910 0.897 **0.924**	0.909 0.913 0.911 0.902 **0.926**	0.910 0.915 0.913 0.905 **0.926**

Bold values means all components gives the best performance.

In **Figure 4B**, the performance comparison of each method in the ablation experiment is shown. Undoubtedly, the performance of the complete process is higher than that of the case without any of the components. In this stepwise experimental result, we find that the information in the note is higher than that in the static indicators. In comparison, the information in the observed data is higher than in the text. If we don’t introduce the instrumental variable, despite using all the data, there is a substantial drop in performance, which means that the model learns the falsity in the observed data and is affected by confounders in the system that are difficult to observe.

## 4 Discussion

**Figure 5:**
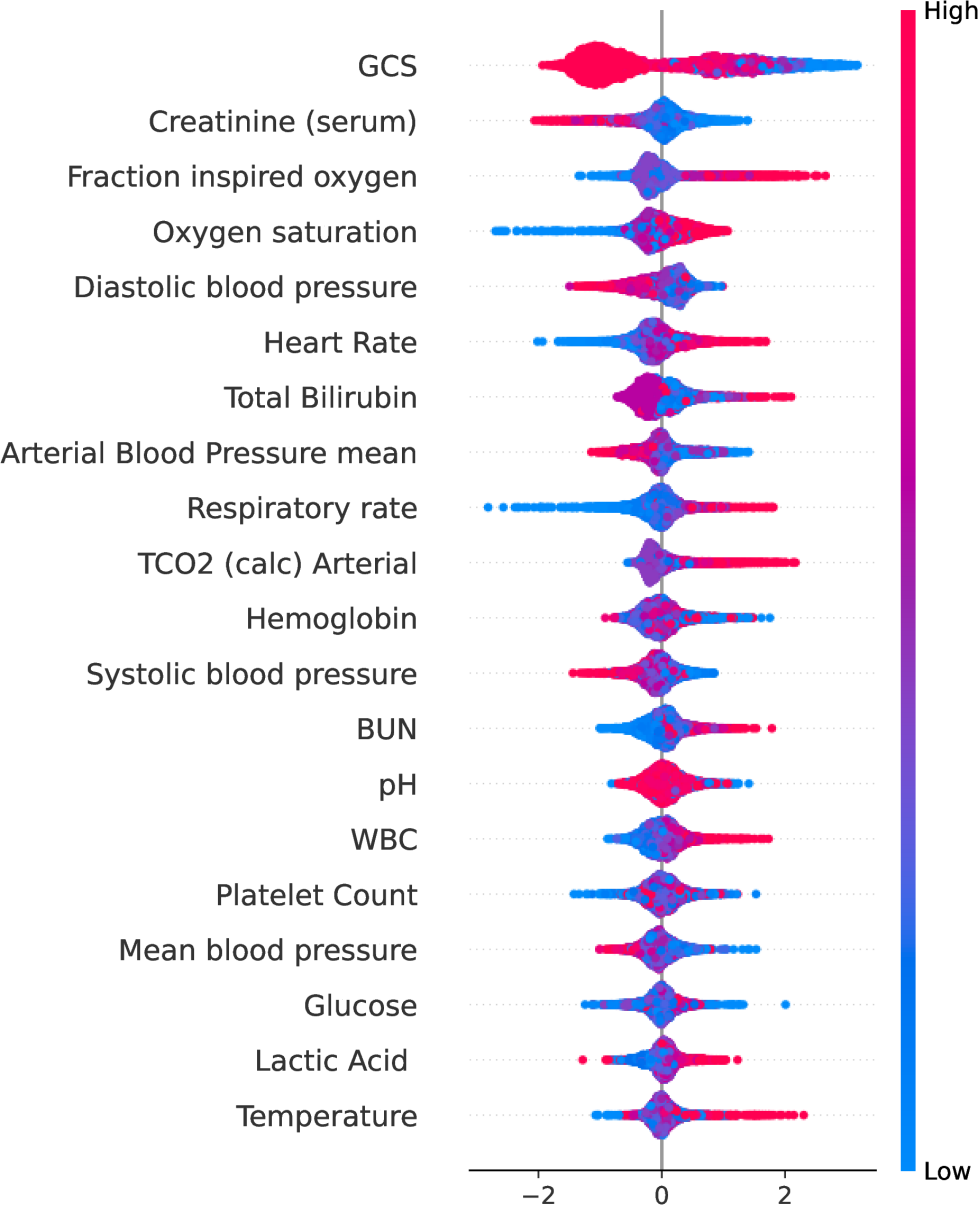
The top twenty variables ranked by importance using SHAP. The ordinate represents the name of each variable in descending order of importance, and the abscissa represents the SHAP value. This plot shows how important each variable is in predicting the final diagnosis of sepsis.

We used the SHAP to calculate the importance of features, as shown in **Figure 5**, which shows the top twenty variables of importance. Here, we plot the Shapley value of each feature for each example. This shows which features are more important and how much impact they have on the dataset. The red feature makes the prediction larger, the blue makes the prediction smaller, and the width of the color region is larger, which means that this feature has a greater impact on the prediction.

Of the top 20 variables, Glasgow coma scale(GCS), Creatinine, Fraction inspired oxygen(FiO2), Total Bilirubin, Arterial Blood Pressure mean and Platelet Count(PC) are part of the SOFA score. In addition, Lactic Acid(LAC) plays a significant role in early disease identification and treatment guidance Rhee et al. (2021). Lactic acidosis refers to an elevated concentration of lactic acid in the bloodstream beyond normal levels, commonly observed in patients with severe sepsis or septic shock Vandewalle et al. (2021). The blood lactic acid level exhibits a positive correlation with sepsis mortality rates Schlapbach et al. (2017). J.R. et al. Moorman et al. (2006) suggest that continuous heart rate monitoring is important in the diagnosis of sepsis in infants in neonatal intensive care units. Early studies indicated that White blood cell count(WBC) serves as an important reference index for assessing sepsis. Karon Karon et al. (2017) and Farkas Farkas (2020) discovered that the sensitivity and specificity of WBC in diagnosing sepsis were 82.2% and 76.7%. Sepsis and septic shock often manifest with an elevated RR, which may serve as a compensatory response to enhance oxygen supply and eliminate excessive carbon dioxide production. Furthermore, sepsis can induce tissue hypoperfusion leading to inadequate oxygenation and hypoxemia, thereby stimulating the respiratory center and ultimately resulting in an increased Respiratory rate(RR) Rhee et al. (2021).

We employ an Instrumental Variable to eliminate the influence of unobservable confounders in the data and utilize Back-door Adjustment to address the impact of observable confounders. By leveraging causal inference, we identify the causal features directly associated with the result and eliminate confounding effects, leading to enhanced performance and generalization capabilities. Promising results have been achieved in early sepsis prediction, which is anticipated to assist physicians in making more accurate judgments before sepsis onset and facilitating timely intervention measures. Notably, note, a potential confounder affecting both causal features and results, has been effectively controlled within our model. However, notes are typically recorded post-event occurrence in practice, which means that the number of notes before the onset of the sepsis is small or doesn’t have useful information. However, the various clinical measures carry most of the information, and the note is only used as additional information to improve the performance of the model. The issue of note needs to be specifically considered for clinical use.

## 5 Conclusion

We propose an early sepsis prediction model based on causal inference, which can effectively avoid spurious correlations and improve model performance and robustness by eliminating the influence of confounding factors to learn true and effective causal relationships. We achieve better performance than baselines on a range of early prediction tasks. The experimental results and subsequent analysis further confirm the effectiveness of our approach, while visualizing some variables that contribute higher to the final prediction. Our study may assist physicians in diagnosing sepsis more accurately before the onset of the disease. It is crucial to predict in advance to arrange and implement the treatment plan for sepsis. Timely treatment can effectively reduce mortality and complications.

## Data Availability

Publicly available datasets were analyzed in this study. This data can be found here: https://github.com/MIT-LCP/mimic-iv.

## References

[B1] Apalak M. Kiasaleh K. (2022). Improving sepsis prediction performance using conditional recurrent adversarial networks. IEEE Access 10, 134466–134476. doi: 10.1109/ACCESS.2022.3230324

[B2] Barton C. Chettipally U. Zhou Y. Jiang Z. Lynn-Palevsky A. Le S.. (2019). Evaluation of a machine learning algorithm for up to 48-hour advance prediction of sepsis using six vital signs. Comput. Biol. Med. 109, 79–84. doi: 10.1016/j.compbiomed.2019.04.027 31035074 PMC6556419

[B3] Bennett A. Kallus N. Schnabel T. (2019). Deep generalized method of moments for instrumental variable analysis. Adv. Neural Inf. Process. Syst. 32, 3564-3574. doi: 10.48550/arXiv.1905.12495

[B4] Chang J. Zhang L. Shao Z. (2023). View-target relation-guided unsupervised 2d image-based 3d model retrieval via transformer. Multimedia. Syst. 29, 3891–3901. doi: 10.1007/s00530-023-01166-y

[B5] Cheng P. Hao W. Dai S. Liu J. Gan Z. Carin L. (2020). “Club: A contrastive log-ratio upper bound of mutual information,” in International conference on machine learning (PMLR). San Diego, CA, United States: JMLR-Journal Machine Learning Research 1779–1788.

[B6] Fagerström J. Bång M. Wilhelms D. Chew M. S. (2019). Lisep lstm: a machine learning algorithm for early detection of septic shock. Sci. Rep. 9, 15132. doi: 10.1038/s41598-019-51219-4 31641162 PMC6805937

[B7] Farkas J. D. (2020). The complete blood count to diagnose septic shock. J. Thorac. Dis. 12, S16. doi: 10.21037/jtd.2019.12.63 32148922 PMC7024748

[B8] Ferrer R. Martin-Loeches I. Phillips G. Osborn T. M. Townsend S. Dellinger R. P.. (2014). Empiric antibiotic treatment reduces mortality in severe sepsis and septic shock from the first hour: results from a guideline-based performance improvement program. Crit. Care Med. 42, 1749–1755. doi: 10.1097/CCM.0000000000000330 24717459

[B9] Gao R.-Y. Jia H.-M. Han Y.-Z. Qian B.-S. You P. Zhang X.-K.. (2022). Calprotectin as a diagnostic marker for sepsis: a meta-analysis. Front. Cell. Infect. Microbiol. 12, 1045636. doi: 10.3389/fcimb.2022.1045636 36519133 PMC9742445

[B10] Goh K. H. Wang L. Yeow A. Y. K. Poh H. Li K. Yeow J. J. L.. (2021). Artificial intelligence in sepsis early prediction and diagnosis using unstructured data in healthcare. Nat. Commun. 12, 711. doi: 10.1038/s41467-021-20910-4 33514699 PMC7846756

[B11] Hartford J. Lewis G. Leyton-Brown K. Taddy M. (2017). “Deep iv: A flexible approach for counterfactual prediction,” in International Conference on Machine Learning (PMLR). San Diego, CA, United States: JMLR-Journal Machine Learning Research 1414–1423. Available online at: https://webofscience.clarivate.cn/wos/alldb/full-record/WOS:000683309501051

[B12] He Y. Xu J. Shang X. Fang X. Gao C. Sun D.. (2022). Clinical characteristics and risk factors associated with icu-acquired infections in sepsis: a retrospective cohort study. Front. Cell. Infect. Microbiol. 12, 962470. doi: 10.3389/fcimb.2022.962470 35967847 PMC9366915

[B13] Hu C. Li L. Huang W. Wu T. Xu Q. Liu J.. (2022). Interpretable machine learning for early prediction of prognosis in sepsis: a discovery and validation study. Infect. Dis. Ther. 11, 1117–1132. doi: 10.1007/s40121-022-00628-6 35399146 PMC9124279

[B14] Inkpen K. Chappidi S. Mallari K. Nushi B. Ramesh D. Michelucci P.. (2023). Advancing human-ai complementarity: The impact of user expertise and algorithmic tuning on joint decision making. ACM Trans. Computer-Human. Interaction. 30, 1–29. doi: 10.1145/3534561

[B15] Jiang S. Zhao D. Wang C. Liu X. Yang Q. Bao X.. (2024). Clinical evaluation of droplet digital pcr in the early identification of suspected sepsis patients in the emergency department: a prospective observational study. Front. Cell. Infect. Microbiol. 14, 1358801. doi: 10.3389/fcimb.2024.1358801 38895732 PMC11183271

[B16] Johnson A. Bulgarelli L. Shen L. Gayles A. Shammout A. Horng S.. (2023). MIMIC-IV, a freely accessible electronic health record dataset. Scientific Data (UK London: Nature Publishing Group) 10, 1. doi: 10.13026/hxp0-hg59 36596836 PMC9810617

[B17] Kaji D. A. Zech J. R. Kim J. S. Cho S. K. Dangayach N. S. Costa A. B.. (2019). An attention based deep learning model of clinical events in the intensive care unit. PloS One 14, e0211057. doi: 10.1371/journal.pone.0211057 30759094 PMC6373907

[B18] Karon B. S. Tolan N. V. Wockenfus A. M. Block D. R. Baumann N. A. Bryant S. C.. (2017). Evaluation of lactate, white blood cell count, neutrophil count, procalcitonin and immature granulocyte count as biomarkers for sepsis in emergency department patients. Clin. Biochem. 50, 956–958. doi: 10.1016/j.clinbiochem.2017.05.014 28552399

[B19] Lauritsen S. M. Kalør M. E. Kongsgaard E. L. Lauritsen K. M. Jørgensen M. J. Lange J.. (2020). Early detection of sepsis utilizing deep learning on electronic health record event sequences. Artif. Intell. Med. 104, 101820. doi: 10.1016/j.artmed.2020.101820 32498999

[B20] Leone M. (2016). Septic shock: A global response. La. Presse. Medicale´. 45, e91–e92. doi: 10.1016/j.lpm.2016.03.002 27079764

[B21] Li Q. Ma H. Song D. Bai Y. Zhao L. Xie K. (2024). “Early prediction of sepsis using chatgpt-generated summaries and structured data,” in Multimedia Tools and Applications. Netherlands: SPRINGERVAN GODEWIJCKSTRAAT. 1–23. Available online at: https://webofscience.clarivate.cn/wos/alldb/full-record/WOS:001157530400003.

[B22] Li J. Wu B. Sun X. Wang Y. (2021). “Causal hidden markov model for time series disease forecasting,” in In Proceedings of the IEEE/CVF Conference on Computer Vision and Pattern Recognition. CA, United States: IEEE., 12105–12114. Available online at: https://webofscience.clarivate.cn/wos/alldb/full-record/WOS:000742075002030

[B23] Liu A.-A. Lu Z. Xu N. Liu M. Yan C. Zheng B.. (2023). Multi-stage reasoning on introspecting and revising bias for visual question answering. ACM Transactions on the Web. New York, NY: ACM.

[B24] Moorman J. Lake D. Griffin M. (2006). Heart rate characteristics monitoring for neonatal sepsis. IEEE Trans. Biomed. Eng. 53, 126–132. doi: 10.1109/TBME.2005.859810 16402612

[B25] Nie W. Yu Y. Zhang C. Song D. Zhao L. Bai Y. (2023). Temporal-spatial correlation attention network for clinical data analysis in intensive care unit. IEEE Trans. Biomed. Eng. 71, 1–14. doi: 10.1109/TBME.2023.3309956 37647192

[B26] Oord A. Li Y. Vinyals O. (2018). Representation learning with contrastive predictive coding. ArXiv. Preprint. arXiv:1807.03748. doi: 10.48550/arXiv.1807.03748

[B27] Pettinati M. J. Chen G. Rajput K. S. Selvaraj N. (2020). “Practical machine learning-based sepsis prediction,” in 2020 42nd Annual International Conference of the IEEE Engineering in Medicine Biology Society (EMBC). New York, NY, USA: IEEE., 4986–4991. doi: 10.1109/EMBC44109.2020.9176323 33019106

[B28] Rhee C. Yu T. Wang R. Kadri S. S. Fram D. Chen H.-C.. (2021). Association between implementation of the severe sepsis and septic shock early management bundle performance measure and outcomes in patients with suspected sepsis in us hospitals. JAMA Netw. Open 4, e2138596–e2138596. doi: 10.1001/jamanetworkopen.2021.38596 34928358 PMC8689388

[B29] Rosnati M. Fortuin V. (2021). Mgp-atttcn: An interpretable machine learning model for the prediction of sepsis. PloS One 16, e0251248. doi: 10.1371/journal.pone.0251248 33961681 PMC8104377

[B30] Schlapbach L. J. MacLaren G. Festa M. Alexander J. Erickson S. Beca J.. (2017). Australian & New Zealand intensive care society (anzics) centre for outcomes & resource evaluation (core) and Australian & New Zealand intensive care society (anzics) paediatric study group: Prediction of pediatric sepsis mortality within 1 h of intensive care admission. Intensive Care Med. 43, 1085–1096. doi: 10.1007/s00134-017-4701-8 28220227

[B31] Singer M. Deutschman C. S. Seymour C. W. Shankar-Hari M. Annane D. Bauer M.. (2016). The third international consensus definitions for sepsis and septic shock (sepsis-3). Jama 315, 801–810. doi: 10.1001/jama.2016.0287 26903338 PMC4968574

[B32] Singh J. Oshiro K. Krishnan R. Sato M. Ohkuma T. Kato N. (2019a). “Utilizing informative missingness for early prediction of sepsis,” in 2019 Computing in Cardiology (CinC) (IEEE), 1–4. https://ieeexplore.ieee.org/document/9005809.

[B33] Singh R. Sahani M. Gretton A. (2019b). “Kernel instrumental variable regression,” in Advances in Neural Information Processing Systems, 32.

[B34] Song D. Yang Y. Li W. Shao Z. Nie W. Li X.. (2024). Adaptive semantic transfer network for unsupervised 2d image-based 3d model retrieval. Comput. Vision Image. Understanding. 238, 103858. doi: 10.1016/j.cviu.2023.103858

[B35] Sun S. Ji W. Tang L. Zhang X. Zhang Y. Feng R. (2022). “An interpretable causal approach for bronchopulmonary dysplasia prediction,” in 2022 IEEE International Conference on Bioinformatics and Biomedicine (BIBM) (IEEE)., 1193–1200. Available online at: https://ieeexplore.ieee.org/document/9995412.

[B36] Vandewalle J. Timmermans S. Paakinaho V. Vancraeynest L. Dewyse L. Vanderhaeghen T.. (2021). Combined glucocorticoid resistance and hyperlactatemia contributes to lethal shock in sepsis. Cell Metab. 33, 1763–1776. doi: 10.1016/j.cmet.2021.07.002 34302744

[B37] Wang Z. Yao B. (2021). Multi-branching temporal convolutional network for sepsis prediction. IEEE J. Biomed. Health Inf. 26, 876–887. doi: 10.1109/JBHI.2021.3092835 34181558

[B38] Yang B. Niu K. Zhu Y. Zheng X. Li T. Wang Z.. (2023). Effects of ondansetron exposure during icu stay on outcomes of critically ill patients with sepsis: a cohort study. Front. Cell. Infect. Microbiol. 13, 1256382. doi: 10.3389/fcimb.2023.1256382 38179420 PMC10764599

[B39] Yuan J. Wu A. Kuang K. Li B. Wu R. Wu F.. (2022). “Auto iv: Counterfactual prediction via automatic instrumental variable decomposition,” in ACM Transactions on Knowledge Discovery from Data (TKDD). New York, NY: Assoc computing machinery, vol. 16, 1–20. Available online at: https://webofscience.clarivate.cn/wos/alldb/full-record/WOS:000804984600014

[B40] Zabihi M. Kiranyaz S. Gabbouj M. (2019). “Sepsis prediction in intensive care unit using ensemble of xgboost models,” in 2019 Computing in Cardiology (CinC) (IEEE), 1. Available online at: https://ieeexplore.ieee.org/document/9005564.

